# TURPxit or not: contemporary management options for benign prostatic obstruction

**DOI:** 10.1007/s00345-021-03780-9

**Published:** 2021-07-20

**Authors:** Thomas R. W. Herrmann, Vincent Misrai, Fernando Gómez Sancha, Thorsten Bach

**Affiliations:** 1 Urology Spital Thurgau AG, Pfaffenholzstrasse 4, 8501 Frauenfeld, Switzerland; 2 Hannover Medical Scholl MHH, Carl Neuberg Str. 1, 30625 Hannover, Germany; 3 Stellenbosch University Western Cape, Stellenbosch, South Africa; 4 Department of Urology, Clinique Pasteur, 45 avenue de Lombez, Toulouse, France; 5 Department of Urology and Robotic Surgery, ICUA-Clínica CEMTRO, Ventisquero de la Condesa 42, 28035 Madrid, Spain; 6 Urology Department, Hill Clinic, Sofia, Bulgaria; 7 Department of Urology, AsklepiosWestklinikumHamburg, Suurheid 20, 22559 Hamburg, Germany

The idea to the header of this special edition of the World Journal of Urology arose during the campaign for the 2019 election in United Kingdom, in a time of uncertainty on the further course of the European Union and its beloved relative from the isle.

This volume is meant to table a motion with regard to the topic of TURPxit. As in Brexit even 2021, it still remains unclear what exactly the TURPxiteers want to leave and want to substitute, furthermore, what the TURPsceptics feel uncomfortable about, and finally, what the remainers want to stay with in the end.

This might probably be because the acronym TURP seems to be descriptive but remains uncertain. To stay within the picture, Brexit makes perfectly sense with regard to the TURP template as the UK and Europe do speak of different matters if they discuss TURP with a PSA reduction of 40% post-TURP for UK [1] and 58% for Europe [2]. The view over the Atlantic which seemed so attractive for the Brexiteers is not providing harmonious consonance as the range for TURP templates goes down to 25% [3]. However, the expectations with regard to a former tempestuous greenish love seem to be different looking into Alex Te’s data [4] with a PSA reduction post-photoselective vaporisation (PVP) of 17%. If one is already happy with that—you definitely have a larger range of potentially satisfying solutions as a surgeon.

This special issue does not operate with divisive tactics, it is targeting that specific group of players who appreciate to have a look on a topic from different angles and find pleasure to hear the arguments of players in a technology-embracing field.

Urology is in love with technology and when it comes to being enamoured with something one still needs to master the evaluation whether it stands up to reality. [5] recently summarised what certainty of evidence is required for the implementation of novel techniques (Table 1). I would like to add to that—as he correctly mentioned that most of the established techniques fell short in providing the evidence before they became gold standards—if the concept/physics of the treatment is sound or if the marketing is just even sounder.

**Table 1:** Requirements for widespread implementation adapted from Speakman MJ et al. [5]

Concept/physics and anatomical considerations are sound
Proof of concept study
Placebo/sham comparison study
Randomised controlled trial against accepted alternative treatment
Cohort studies: to understand the generalisability and potential harms
Systematic reviews and meta-analysis of high-quality primary studies
How many patients should be included?—a sample size determination is needed
What should be the length of follow-up?
Short term: < 12 months, medium 12–36 months and long term > 36 months
Inclusion and exclusion criteria need to allow good generalisability
Relevant outcomes—varied and need to be clearly outlined from the start

With regard to TURPxit, if you want to leave your old love, you should better examine the matter very carefully once again, and Räto Strebel and Steven Kaplan [6] have done this elegantly as the introduction to this issue. Even if the love for green seems to fade a little, the article of Kevin Zorn an colleagues [7] give an overview of the benefits of PVP vaporisation. As in any other field the enemy of what you might consider good is better, this is why Vincent Misrai and colleagues [8] and colleagues provided a comparison of vaporisation vs. enucleation by recontextualising the green light.

Sometimes reasons beyond first site make your old love look more attractive if you set up new objectives as the main goal—away from durability. The topic in focus is the expectation of unchanged sexual function and namely antegrade ejaculation. Jean de la Rosette and colleagues [9] have added articles which scrutineers whether ejaculation is a valid endpoint or a conundrum. Although this manuscript is published online since 1st April, this article touches the core of relevance and probably reasons for existence in the nearer and further future of all so called new minimal invasive technologies (MIS).

Aquablation as a real-time image-guided therapy has gained substantial attention in the last years. Kevin Zorn and many fathers [10] of aquablation provide a summary on what the truth there is in this technology, today.

Although the results of multi-institutional UK-ROPE study [11] poured some acid in the wine of new shores of prostate arterial embolisation, Dominik Abt, Hans Peter Schmid and Mark Speakman [12] shed a light on the potential of this technique in their article.

If aquablation comes closest to what technology can achieve in future combined with artificial intelligence, the other aspired technique of today seems to keep the treatment in the hand of the steampunk prone surgeon in 2021. The available data off-site RCT against surgical comparator are explained by the third paper from Kevin Zorn and colleagues [13]. The group around Christian Gratzke [14] is summarising the rationale and evidence for the new MIS, namely iTIND, Urolift and Rezūm besides the shortcomings mentioned by Mark Speakerman in the above-mentioned article on certainty.

Robotic surgery is always in front when it comes to total procedural costs and total cost of investment. Therefore, it is in question whether in lack of RCT it should have a position aside anatomical enucleation of the prostate AEEP, as it clearly follows the principal of anatomical enucleation. In their article, Hubert John and Christian Pandevit from Winterthur as well as Christian Wagner and Jörn Witt from Gronau have [15] come to the conclusion that the time is right and provide a nice overview on robotic (simple) adenomectomy RASP.

The discussion on MIS of the 2016 till today mirrors in some aspects the discussion of the 2007 till 2016. The mayor difference is the anatomical clearly defined template on side of the AEEP and the extrananatomical template, which can be gradually modified on the side of the novel MIS.

The field of AEEP which has been pacified by the Magna Charta of AEEP (all anatomical enucleations are equally effective [16] has produced some interesting discussions with regard to superiority to other treatments on one hand and safety with regard to early functional results on the other, especially in the context of learning curve [17] [18].

The ground for the strife for better results is the preservation of intrasphincteral mucosa [19] and the influence of en bloc preparation vs two lobe or three-lobe technique in contemporary AEEP. An article penned from the group around one of the most experienced HoLEP Surgeons in the world, Karin Lehrich and colleagues [20] shed light on the topic of which seems to be—lobe wise—the best strategy after an estimated cumulative experience of more than 10,000 HoLEPs of the group of authors.

Tev Aho and colleagues [21] provided two articles on selected group of patients in AEEP, namely HOLEP in this case. The effectivity of AEEP as maximum deobstruction in patients with non-neurogenic chronic urinary retention seems to be ideally suited for patients with this condition. The other article about extra large prostates is pacing around the front line towards RASP, [22].

With the introduction of novel Thulium lasers that change the perception of Thulium in stone and soft tissue surgery Benedict Becker [23] and colleagues summarise the evidence for Thulium in AEEP in the light of new Thulium generators, be it fibre laser or novel YAG lasers. Since 2008, the debate whether Holmium or Thulium is the greater asset when it comes to AEEP is open. The group of Giorgio Bozzini [24] added another piece of information providing the data from a multicenter RCT comparing HoLEP with ThuLEP.

The data from bipolar enucleation have paved the way towards the overarching principle of AEEP in 2016 [25], [26]. As stated above, the quest for best energy source for each signature patients in AEEP is an ongoing debate. Lukas Lusuardi and colleagues [27] are revisiting that discussion in a systematic review of EEP—same but different.

The special issue of TURPxit or not—contemporary management options for benign prostatic obstruction ends with three personal articles. The first by Stavros Gravas, EAU Male Luts Guidelines chairman, [28] cast a view on the endourology as a field in motion thereby being endowed with the serenity of Greek philosophy of πάντα ῥεῖ. The second last editorial is contributed by guest editor [29] Fernando Gomez Sancha, highlighting quest from TURP to the best possible treatment for BPO–AEEP.

And last but not least as a closing remark: surgeon’s heuristic and decision making in the face of all of the above displayed treatment options by guest editors Vincent Misrai and Thomas RW Herrmann [30].

We would like to cordially thank all contributors for the tireless efforts to enable such a nice collection of articles.

We wish all of you a pleasant reading of this special issue—which does not call for a general election of the best treatment, nor seeks a hard border in between the treatment option but keeps us united as endourological surgeons wanting to provide the best options for our patients.
